# Neurochemicals Involved in Medullary Control of Common Carotid Blood Flow

**DOI:** 10.2174/1570159X113119990044

**Published:** 2013-09

**Authors:** Chi-Li Gong, Yuk-Man Leung, Ming-Ren Wang, Nai-Nu Lin, Tony Jer-Fu Lee, Jon-Son Kuo

**Affiliations:** 1 Department of Physiology, School of Medicine, China Medical University, Taiwan;; 2 Graduate Institute of Neural and Cognitive Sciences, China Medical University, Taiwan;; 3 Yuhing Junior College of Health Care and Management, Kaohsiung, Taiwan;; 4 Department of Education and Research, Taichung Veterans General Hospital, Taichung, Taiwan;; 5 Neuro-Medical Scientific Center and Center for Vascular Medicine, Buddhist Tzu Chi General Hospital and Tzu Chi University, Hualien, Taiwan;; 6 Department of Medical Research, Buddhist Tzu Chi General Hospital and Tzu Chi University, Hualien, Taiwan;; 7 Institute of Pharmacology and Toxicology, Tzu Chi University, Hualien, Taiwan

**Keywords:** Carotid artery, Cerebral blood flow, Medulla, Parasympathetic nucleus, Vascular regulation, Neurotransmitter

## Abstract

The common carotid artery (CCA) supplies intra- and extra-cranial vascular beds. An area in the medulla controlling CCA blood flow is defined as the dorsal facial area (DFA) by Kuo et al. in 1987. In the DFA, presynaptic nitrergic and/or glutamatergic fibers innervate preganglionic nitrergic and/or cholinergic neurons which give rise to the preganglionic fibers of the parasympathetic 7th and 9th cranial nerves. Released glutamate from presynaptic nitrergic and/or glutamatergic fibers can activate N-methyl-D-aspartate (NMDA) and α-amino-3-hydroxy-5-methylisoxazole-4-propionic acid (AMPA) receptors on preganglionic nitrergic and/or cholinergic neurons. By modulating this glutamate release, several neurochemicals including serotonin, arginine, nitric oxide, nicotine, choline and ATP in the DFA regulate CCA blood flow. Understanding the neurochemical regulatory mechanisms can provide important insights of the physiological roles of the DFA, and may help develop therapeutic strategies for diseases involving CCA blood flow, such as migraine, hypertensive disease, Alzheimer’s disease and cerebral ischemic stroke.

## INTRODUCTION

Common carotid artery (CCA) is an important artery supplying both intra- and extra-cranial tissues of the head, but the brain regulation of CCA blood flow has been rarely addressed. Vasodilatations in extra- and intra-cranial tissues have long been known to be induced by stimulating parasympathetic 5^th^ [1, 2], 7^th^ and 9^th^ cranial nerves or by stimulating the locus coeruleus or nucleus raphe dorsalis [3, 4]. In addition, CCA blood flow increase that involves in the defense reaction can be induced by stimulations of the periaqueductal gray of the midbrain [5, 6]. However, a preganglionic nucleus controlling CCA blood flow had not been investigated until Kuo *et al.* reported that dorsal portion of the lateral tegmental field in cat medulla controlled mainly an ipsilateral increase in CCA blood flow without significant changes in other cardiovascular parameters [7]. Thence the authors defined this area as the dorsal facial area (DFA) because it is located dorsally to the facial nucleus in cats. Similar to the location of DFA, an area located dorsolaterally to the facial nucleus in rats was defined by Nakai *et al*. [8] as a parasympathetic cerebrovasodilator center.

In the last 25 years, we have established anatomical organizations and several neurochemical regulatory mechanisms of the DFA, as summarized in Figs. (**1** and **2**). Understanding the anatomical organizations and neuro-chemical regulatory mechanisms may provide important information for understanding the physiological functions of the DFA and developing therapeutic strategy for diseases involving CCA blood flow, such as migraine, hypertensive disease, Alzheimer’s disease, and cerebral ischemic stroke. 

## DFA IS A PARASYMPATHETIC PREGANGLIONIC NUCLEUS

DFA-induced increase in CCA blood flow is abolished by ipsilateral sectioning of both parasympathetic 7^th^ and 9^th^ cranial nerves, but is not abolished by ipsilateral cervical sympathectomy [7]. It is partially blocked by intravenous administrations of high dose of atropine, a parasympathetic blocking agent [9]. In addition, ChAT-reactive neurons (cholinergic neurons) are present in the DFA where preganglionic neurons give rise to the parasympathetic 7^th^ and 9^th^ cranial nerves and they seem to be colocalized in the inferior and superior salivary nuclei [10]. The DFA of the cat may be functionally and anatomically equivalent to an area located dorsolaterally to the facial nucleus in rats as demonstrated by Nakai *et al*. [8] who defined the area as a parasympathetic cerebrovasodilator center. Based on the anatomical location, both areas in cats and rats are likely the rostral extension of the dorsal motor nucleus of the vagus nerve. The DFA is therefore a parasympathetic preganglionic nucleus that gives rise to both parasympathetic 7^th^ and 9^th^ cranial nerves (Fig. (**1**)). 

## DIFFERENTIAL CONTROL OF INTRA- AND EXTRA-CRANIAL BLOOD FLOWS

Since CCA supplies blood flows to intra- and extra-cranial tissues, while the DFA-induced CCA blood flow increase can only be partially blocked by intravenous administration of atropine, indicating different mechanisms for increasing blood flows in intra- and extra-cranial vessels [9]. The increase in extra-cranial tissues is completely abolished by atropine but further increased by physostigmine (an acetylcholine-esterase inhibitor acting as a muscarinic agonist), demonstrating that the increase in extra-cranial tissues is completely mediated by muscarinic action of acetylcholine. The increase in intra-cranial tissues, in contrast, is enhanced by atropine but inhibited by physostigmine, suggesting that the increase in intra-cranial tissues is mediated by a non-muscarinic vasodilator mechanism (Fig. (**1**)); nevertheless, this finding also indicates that a muscarinic mechanism can inhibit the increase in intra-cranial tissues. The non-muscarinic vasodilator for the increase in intra-cranial tissues, possibly a co-transmitter nitric oxide, can be released in association with the release of acetylcholine to the brain vessel from the post-ganglionic fibers of parasympathetic 7^th^ and 9^th^ cranial nerves and be modulated by the axo-axonal mechanism for cerebral vasomotor regulations [11, 12]. The muscarinic inhibition to the increase in intra-cranial tissues may be attributed to an inhibition by muscarinic action of acetylcholine to the vasodilator effect of adrenergic *β*-receptor on the parasympathetic terminal. This mechanism explains the notion that muscarinic action can inhibit the increase in the intra-cranial blood flow induced by DFA stimulation [9].

In conclusion, DFA stimulation promotes release of acetylcholine to CCA vascular beds, the vessels of the intra- and extra-cranial tissues. The muscarinic receptor is responsible for increasing blood flow in the extra-cranial tissues, but for inhibiting the increase of blood flow in the intra-cranial tissues. For increasing intra-cranial blood flow, the non-cholinergic vasodilator is most likely nitric oxide co-released with acetylcholine from the parasympathetic terminals, which are modulated by the axo-axonal mechanism. 

## GLUTAMATERGIC ACTION, RELEASE AND RECEPTORS

An increase in CCA blood flow evoked by glutamate stimulation of the DFA was first reported by Kuo *et al*. [7]. This response is independent of the higher center, since the response is not abolished by supra-collicular decerebration [10]. Activation of the DFA with glutamate, N-methyl-D-aspartate (NMDA) and α-amino-3-hydroxy-5- methylisoxazole-4-propionic acid (AMPA) induces dose-dependent increases in CCA blood flow, and the order of potencies is AMPA>NMDA>glutamate [13]. Pretreatment with either non-competitive NMDA receptor antagonist or AMPA/kainate receptor antagonist attenuates the glutamate-induced increase in CCA blood flow in a dose-dependent manner [13]. These findings demonstrate that NMDA and AMPA receptors on neurons in the DFA are responsible for the increase in CCA blood flow. 

The microdialysis hyphenated with high performance lipid chromatography (HPLC) [14] is a useful technique for measuring glutamate and serotonin (5-HT) released in the brain [15-17]. Perfusion with KCl, a neuronal depolarizing agent, through a microdialysis probe in the DFA increases dose-dependently glutamate concentration in the dialysate, suggesting an endogenous release of glutamate in the DFA [15]. Perfusion with 5-HT or alaproclate (a serotonin reuptake inhibitor) decreases glutamate concentration accompanied with a parallel decrease in CCA blood flow, suggesting a tonic glutamate release is inhibited by a tonic5-HT release in the DFA [15]. The latter proposal was confirmed later [13, 16]. Neuronal release of glutamate is further confirmed by the findings that CCA blood flow increase induced by intra-DFA administrations of several neurochemicals, such as arginine (NO precursor), S-nitroso-N-acetylpenicillamine (NO donor) [18], ATP (P2 receptor agonist) [19], and choline (α7-nAChR agonist) or nicotine (non-specific nAChR agonist), can be inhibited by glutamatergic receptor antagonists. Furthermore, endo-genously released [18] and exogenously administered glutamate [13] are similarly blocked by the NMDA or AMPA receptor antagonist; both receptors have therefore been proposed to be present on preganglionic neurons, nitrergic and/or cholinergic neurons which give rise to the parasympathetic 7^th^ and 9^th^ preganglionic nerves [18].

In conclusion, the glutamatergic fiber releasing glutamate in a tonic manner is present in the DFA. The released glutamate stimulates both AMPA and NMDA receptors on the preganglionic neurons in the DFA to induce an increase of CCA blood flow. Various neurochemicals may activate their respective receptors on the glutamatergic fiber to modulate the glutamate release in the DFA. 

## SEROTONERGIC RELEASE, ACTION, AND RECEPTOR

The 5-HT nerves and their receptors exist widely in the brain including the medulla oblongata, playing important roles in cardiovascular or cardiopulmonary regulations. For example, the 5-HT_2_ receptors in the nucleus ambiguous mediates GABAergic activity [20]; the 5-HT_2A_ receptor in the nucleus tractus solitarious inhibits sympathetic activity [21] and the 5-HT_3_ and 5-HT_4_ receptors in the nucleus tractus solitarii participate in the cardiopulmonary reflex [22]. Furthermore, activation of various brain regions with selective agonists for 5-HT_1D_, 5-HT_2_, and 5-HT_2A_ receptors or with 5-HT reuptake inhibitors can inhibit glutamate release [15, 23-25]. Nevertheless, Li *et al*. [15] first reported 5-HT inhibition of glutamate release in the DFA. 

5-HT-reactive nerves in the DFA have been identified by immunohistochemical staining of tyrosine hydroxylase [10, 26]. These anatomical findings are functionally substantiated by a finding of neuronal release of 5-HT in tonic [9]. Furthermore, perfusion in the DFA with 5-HT_2_ receptor antagonist increases while that with 5-HT_2_ receptor agonist decreases glutamate release in the DFA, accompanied with an increase and a decrease of CCA blood flow [15], respectively. Perfusion with 5-HT_1_ agonist or antagonist, however, does not affect glutamate release in the DFA [16]. 5-HT_2_ but not 5-HT_1_ action in the DFA is further substantiated by pharmacological interactions of various agonists and antagonists for 5-HT_1_ and 5-HT_2 _in the DFA [13]. 

In conclusion, 5-HT reactive nerves are present in the DFA; they release 5-HT in tonic, which activates 5-HT_2_ receptors on the presynaptic nitrergic and/or glutamatergic fibers to cause an inhibition of glutamate release in the DFA, leading to the reduction in CCA blood flow. 

## NICOTINIC ACTIONS AND RECEPTORS

Wide cholinergic innervations and diverse muscarinic and nicotinic actions are present in the brain and medulla for cardiovascular regulations or transmitter releases. For instance, activation of cholinergic nerves projecting to the rostral ventrolateral medulla [27], the dorsal motor nucleus and solitary nucleus of the vagus nerve [28] can regulate various cardiovascular functions. The α7-nAChR on the striatal glutamatergic terminals promotes glutamate release [29]. The α3β2- and α4β2-nAChRs on dopaminergic terminals in the striatum promote dopamine releases [29]. Nevertheless, Gong *et al*. [30] demonstrated for the first time that activation of α7-, α4β2-, and α3β4-nAChRs in the DFA increase CCA blood flow, and Kuo *et al*. [31] further demonstrated that activation of α7-nAChR on glutamatergic terminals in the DFA leads to glutamate release, as evidenced by the following experiments. 

Nicotine (a non-selective nAChR agonist) or choline (a selective α7-nAChR agonist) stimulation of the DFA results in dose-dependent increases in CCA blood flow [30]. The nicotine-induced increase is dose-dependently inhibited by α7-nAChR antagonists (α-bungarotoxin and methyllycaconitine), a relatively selective α4β2-nAChR antagonist (dihydro-β-erythroidine), and a relatively selective α3β4-nAChRs antagonist (mecamylamine), while the choline-induced increase is inhibited in a large extent by α7- and, in a lesser extent, by α3β4 nAChR antagonist, but not by α4β2-nAChRs antagonist, indicating α7-nAChR plays the most important role [30]. Furthermore, microinjections of muscarinic receptor agonists (muscarine and methacholine) and antagonist (atropine) do not affect the basal CCA blood flow; muscarinic receptors if any in the DFA, therefore, are not likely to regulate CCA blood flow. These findings suggest the presence of α7 (the major subtypes), α4β2, and α3β4 subunits of nAChRs in the DFA and their involvement in regulation of CCA blood flow.

In another experiment, the increase of CCA blood flow induced by stimulation of the DFA with nicotine or choline is abolished by pretreatment with either α7-nACh receptor antagonist (α-bungarotoxin) or glutamate antagonist (such as MK-801, an NMDA antagonist, or glutamate diethylester, an AMPA antagonist), suggesting glutamate release in the DFA upon activation of α7-nACh receptor by nicotine or choline [31]. On the other hand, the flow increase induced by intra-DFA administration of glutamate or KCl, which can induce releases of various transmitters including glutamate and acetylcholine, is greatly inhibited by MK-801 and glutamate diethylester, but not by α-bungarotoxin [31]. These findings suggest that activation by nicotine or choline of nACh receptors, primarily α7-nACh receptors, causes a release of glutamate, but does not cause a release of cholinergic substance that stimulates α7-nACh receptors in the DFA. The α7-nAChR and other nAChRs may be present on the nitrergic and/or glutamatergic fiber which innervates and releases glutamate to stimulate preganglionic neurons of the parasympathetic 7^th^ and 9^th^ cranial nerves, leading to an increase in CCA blood flow [18].

In conclusion, functional α7, α4β2, and α3β4 subunits of nAChRs, with the most prominent one being the α7 subunit, appear to be present in the DFA on the nitrergic and/or glutamatergic fiber which innervates and releases glutamate to stimulate the preganglionic neurons of the parasympathetic 7^th^ and 9^th^ cranial nerves for increasing CCA blood flow. Muscarinic receptors if any in the DFA are not likely to regulate CCA blood flow. 

## PURINERGIC AGONISTS AND RECEPTORS

Although adenosine (a P1 purinergic receptor agonist) is generated by enzymatic degradation of the neuro-gliotransmitter ATP, adenosine is exocytotically released [32, 33] in the brain affecting cardiovascular function. Adenosine modulates neuron activities in the rostral ventrolateral medulla [34] and baroreceptor activities in the area postrema [35] and the nucleus tractus solitarii [36]. On the other hand, ATP triggers physiological functions *via *activating P2 purinoceptors that are divided into two main families: P2X and P2Y purinoceptor families [37]. ATP in the medulla regulates cardiovascular functions [38] through modulating neuron activities *via *purinergic actions [39, 40]. Furthermore, ATP can cause release of glutamate through stimulating purinergic receptors in the brain stem autonomic network [41].

An assertion that adenosine and ATP can mediate release of glutamate through stimulating purinergic receptors in the DFA to increase CCA blood flow has been demonstrated by Kuo *et al* [19]. Microinjection into the DFA with adenosine, a P1 receptor agonist, results in only mild and poorly reproducible increase, while stimulation with ATP or α,β-MeATP, a P2 purinergic receptor agonist, results in a markedly dose-dependent increase in CCA blood flow. P2 receptor-induced increase in CCA blood flow is dose-dependently attenuated by pretreatment with either P1 receptor antagonist (dipropyl-8-p-sulfophenylxanthine, DPSPX) or P2 receptor antagonist (pyridoxalphosphate-6-azophenyl-2’, 4’-disolfonic acid, PPADS). The effect of ATP or α,β-MeATP, a P2 receptor agonist, is also inhibited by P1 receptor antagonist, suggesting a degradation of ATP to adenosine, a P1 receptor agonist. The increase of CCA blood flow caused by purinergic agonists (ATP and α,β-MeATP) or glutamate is dose-dependently attenuated by pretreatment with MK-801 (a non-competitive NMDA receptor antagonist) or glutamate diethyl ester (GDEE, a competitive AMPA/kainite receptor antagonist), indicating that the purinergic activation mediates a release of glutamate that stimulates AMPA and NMDA receptors in the DFA to induce the increase in CCA blood flow. The above findings suggest that P2 and P1 purinergic receptors are present in the DFA, with P2 receptors being the majority; activation of these two receptors result in release of glutamate, which increases CCA blood flow. 

In conclusion, purinergic receptors, predominantly P2 with a lesser extent P1, appear to be present in the DFA; activations of these receptors result in release of glutamate that activates the NMDA and AMPA receptors in the DFA, leading to the increase in CCA blood flow.

## PRESYNAPTIC NITRERGIC AND/OR GLUTA-MATERGIC FIBER 

Nitric oxide synthases (NOSs), which in the presence of Ca^2+^-calmodulin catalyze L-arginine (a NO precursor) to synthesize NO in cells, include neuronal NOS (nNOS), endothelial NOS (eNOS), and inducible NOS (iNOS). The nitrergic cells/neurons (the NOS-containing neurons) releasing NO as a neurotransmitter are widely distributed in the brainstem [42, 43], including the nucleus tractus solitarius [44, 45], hypothalamus [46], and rostral ventrolateral medulla [47]. The nNOS, nevertheless, is co-localized in the glutamatergic neurons/fibers in the rostral ventrolateral medulla [48]. This suggests that NO, synthesized by nNOS in these neurons/fibers, may be released to act as a neurotransmitter itself, or alternatively may stimulate guanylyl cyclase to convert cyclic guanosine triphosphate (cGTP) to cyclic guanosine monophosphate (cGMP) that may then stimulate release of glutamate from the glutamatergic neurons/fibers. The latter notion is seldom addressed except for a report in which presynaptic nitrergic and/or glutamatergic fibers have been proposed to be present in the solitary nucleus, releasing NO and glutamate in the regulation of sympathetic out flow [49]. Similar presynaptic nitrergic and/or glutamatergic fibers have been proposed to be present in the DFA in the regulation of CCA blood flow [18, 50] according to the following findings.

Profuse nitrergic fibers surrounding nitrergic neurons in the DFA have been identified with nicotinamide adenine dinucleotide phosphate diaphorase (NADPH-diaphorase), a marker for the nitrergic neurons [50]. The increased CCA blood flow induced by intra-DFA administration of L-arginine is markedly inhibited not only by pretreatment with nNOS inhibitor but also by glutamate receptor antagonists, suggesting that nNOS-containing neurons/fibers may release glutamate in the DFA [50]. In microdialysis and HPLC studies, perfusion with a NO donor into the DFA increases, but co-perfusion of a NO donor with a guanylyl cyclase (GC) inhibitor blocks release of glutamate, further suggesting that glutamate is released from the nNOS/GC/cGMP-containing neuron/fiber in the DFA and glutamate release is triggered by cGMP [18]. The above findings suggest that the presynaptic glutamatergic fiber in the DFA can release glutamate through activation of the nNOS/GC/cGMP pathway in the fiber, and is defined as presynaptic nitrergic and/or glutamatergic fibers. The released glutamate thus activates post-synaptically on the preganglionic neurons of parasympathetic 7^th^ and 9^th^ cranial nerves (Fig. (**2**)).

In conclusion, the presynaptic nitrergic and/or glutamatergic fibers that can release glutamate through activation of the nNOS/GC/cGMP in the fiber exist in the DFA for regulation of CCA blood flow [18].

## PREGANGLIONIC NITRERGIC AND/OR CHOLINERGIC NEURON 

Both cholinergic neurons [10, 26] and nitrergic neurons [50] are present in the DFA. Preganglionic neurons projecting to the parasympathetic sphenopalatine ganglia of the facial nerve reside also in the DFA [10]. All these neurons are similar in morphology and location in the DFA, indicating that the preganglionic neurons are nitrergic and cholinergic in nature. Thus they are named preganglionic nitrergic and/or cholinergic neuron [18]. Other studies demonstrate that glutamate exogenously administered [13], or endogenously released from the presynaptic nitrergic and/or glutamatergic fibers [18] in the DFA causes the increase in CCA blood flow, which can be markedly inhibited by nNOS or guanylyl cyclase (GC) inhibitors as well as glutamatergic (NMDA and AMPA) receptor inhibitors. The above findings together suggest that the glutamate released from the presynaptic nitrergic and/or glutamatergic fibers may stimulate the NMDA and AMPA receptors on the preganglionic nitrergic and/or cholinergic neuron.

In conclusion, glutamate-releasing presynaptic nitrergic and/or glutamatergic fibers innervate preganglionic nitrergic and/or cholinergic neurons, which bear NMDA and AMPA receptors for glutamate [18] (Fig. (**2**)).

## NEUROCHEMICAL MODULATIONS OF PRE-SYNAPTIC NITRERGIC AND/OR GLUTAMATERGIC FIBERS BY NNOS/GC/CGMP SYSTEM

Glutamate release by the presynaptic nitrergic and/or glutamatergic fibers in the DFA depends on activation of the nNOS/GC/cGMP system in the presynaptic nitrergic and/or glutamatergic fibers [18, 50, 51]. These findings suggest that neurochemical modulations through their respective receptors on the presynaptic nitrergic and/or glutamatergic fibers to release glutamate can be mediated *via *the activations of the nNOS/GC/cGMP system. This hypothesis has been supported by the following findings. 

Intra-DFA administrations of arginine (NO precursor) or S-nitroso-N-acetylpenicillamine (NO donor) [18, 50], choline, nicotine [31, 51] or ATP [19] in the DFA induce an increase of CCA blood flow. The increased CCA blood flow can be blocked by the antagonist for glutamate or the nNOS/GC/cGMP system, suggesting glutamate release *via *activation of the nNOS/GC/cGMP system in the presynaptic nitrergic and/or glutamatergic fibers. The increased CCA blood flow can also be blocked by the respective receptor antagonists for the above mentioned neurochemicals, suggesting a neurochemically presynaptic activation of the presynaptic nitrergic and/or glutamatergic fibers to release glutamate is mediated *via *activation of the nNOS/GC/cGMP system in the presynaptic nitrergic and/or glutamatergic fibers. 

In conclusion, neurochemical agonists may presynaptically act on their respective receptors on the presynaptic nitrergic and/or glutamatergic fibers to activate the nNOS/GC/cGMP system and subsequently modulate glutamate release (Refer to Fig. (**2**)). 

## Figures and Tables

**Fig. (1):**
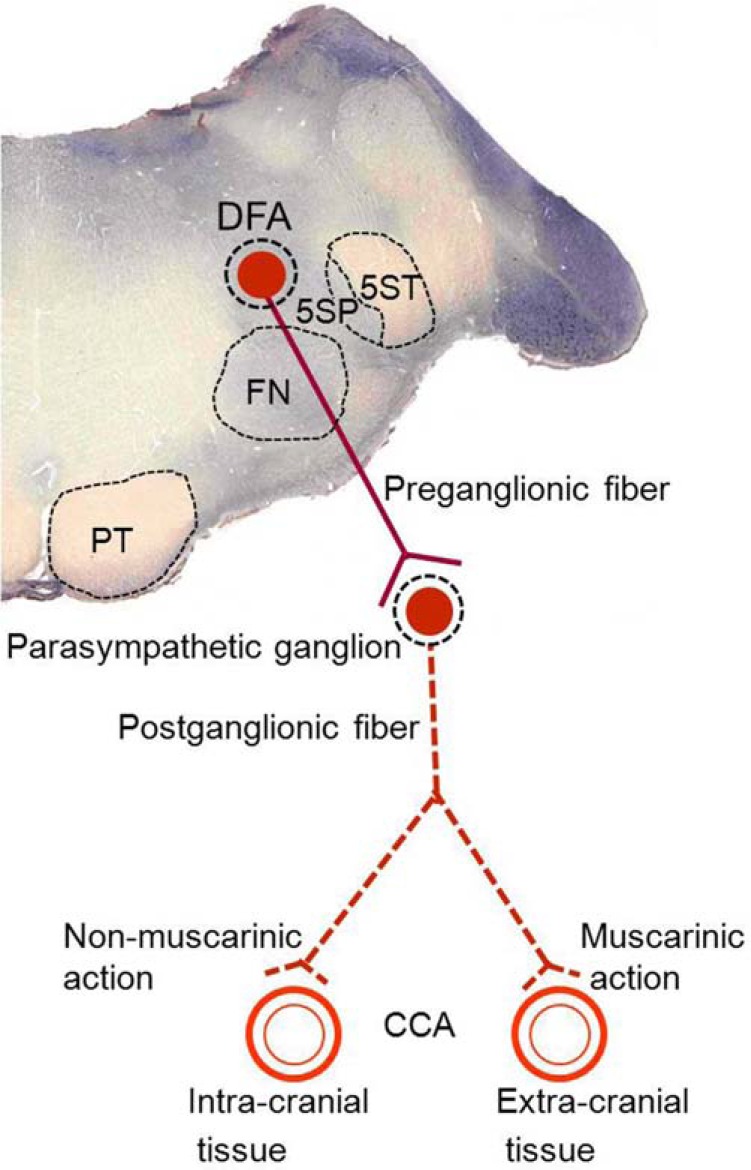
The DFA and its related connections. The DFA is located at the medulla dorsally to the facial nucleus. The preganglionic neurons in the DFA give rise to the preganglionic and postganglionic fibers of the parasympathetic 7^th^ and 9^th^ cranial nerves. The CCA vascular beds that supply intra- and extra-cranial tissues are innervated by the postganglionic fiber that releases acetylcholine. Muscarinic and nonmuscarinic action of acetylcholine is responsible for blood flow increase in the extra-cranial and intra-cranial tissues, respectively. Abbreviations: CCA, common carotid artery; DFA, dorsal facial area; FN, facial nucleus; 5SP, spinal trigeminal nucleus; 5ST, spinal trigeminal tract; PT, pyramidal tract.

**Fig. (2):**
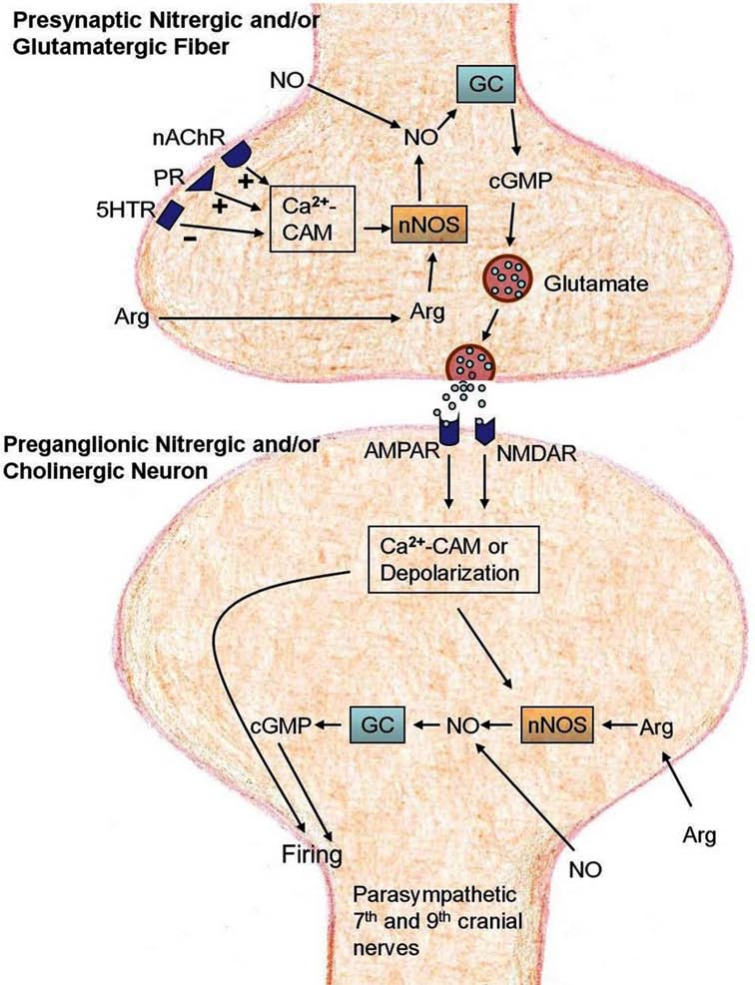
Neurochemical modulations in the DFA. Preganglionic nitrergic and/or glutamatergic fiber and preganglionic nitrergic and/or cholinergic neurons contain the nNOS/GC/cGMP system. Nitric oxide (NO) and arginine may presynaptically activate the nNOS/GC/cGMP system in the preganglionic nitrergic and/or glutamatergic fiber to induce glutamate release, or postsynaptically activate that in the preganglionic nitrergic and/or cholinergic neurons to induce neuron firing (excitation). Other neurochemicals have their receptors on the preganglionic nitrergic and/or glutamatergic fiber but not on the preganglionic nitrergic and/or cholinergic neurons. Choline and nicotine may presynaptically activate nAChRs, while ATP and adenosine activate PR on the preganglionic nitrergic and/or glutamatergic fiber to enhance the nNOS/GC/cGMP activities in the fiber, resulting in glutamate release; nicotinic or choline action is mediated primarily *via* α7-nAChR, and purine/ATP action primarily *via* P2 receptor. However, 5-HT stimulation of 5HTR on the presynaptic nitrergic and/or glutamatergic fibers is mediated primarily by 5-HT2 receptor, probably through an inhibition of the nNOS/GC/cGMP activities leading to a reduction of glutamate release. The released glutamate in turn postsynaptically activates NMDA and AMPA receptors on the preganglionic nitrergic and/or cholinergic neurons. This activation may directly depolarize these neurons *via* NMDA or AMPA channels and/or indirectly depolarize preganglionic nitrergic and/or cholinergic neurons *via* activating their nNOS/GC/cGMP system. Consequently excitation impulses of the preganglionic nitrergic and/or cholinergic neurons are conducted through the pre- and post-ganglionic fibers of the parasympathetic 7^th^ and 9^th^ cranial nerves to CCA vascular beds for intra- and extra-cranial tissues, causing increase of blood flow in these tissues. Abbreviations: AMPAR, receptor for α-amino-3-hydroxy-5- methylisoxazole-4-propionic acid; Arg, L-arginine; CAM, calmodulin; cGMP, cyclic guanosine monophosphate; 5HTR, serotonin receptor; GC, guanylyl cyclase; nAChR, nicotinic acetylcholine receptor; NMDAR, receptor for N-methyl- D-aspartate; NO, nitric oxide; nNOS, neuronal NO synthase; PR, purinergic receptor.

## CONCLUDING REMARKS

Both presynaptic nitrergic and/or glutamatergic fibers and preganglionic nitrergic and/or cholinergic neurons are activated through the nNOS/GC/cGMP system in the DFA. The released glutamate from presynaptic nitrergic and/or glutamatergic fibers can activate the preganglionic nitrergic and/or cholinergic neurons which bear the NMDA and AMPA receptors and give rise to the preganglionic fiber of the parasympathetic 7th an 9th cranial nerves controlling CCA blood flow. The release of glutamate from presynaptic nitrergic and/or glutamatergic fibers can be modulated by many neurochemicals, neurotransmitters, and neuro-modulators, such as arginine (NO precursor) or S-nitroso-N-acetylpenicillamine (NO donor), serotonin, choline, nicotine, ATP, or even more to be addressed in the future. All these mechanisms integrate to play important roles in regulation of CCA blood flow that supplies the intra- and extra-cranial tissues. Presence of various receptors of neurochemicals, neurotransmitters, and neuromodulators in the DFA may produce physiological or pathological effects that regulate intra- and extra-cranial circulation.

So far as we know, “the role of the DFA in pathophysiology associated with altered carotid blood flow” has not been investigated. It is interesting and possible that many factors such as smoking (effects of nicotine), glutamate in food, neural inflammation, neural degeneration in aging, agonists and antagonists of serotonin, and purine, may involve in the pathophysiology of the DFA. Although the pathological role of the DFA is yet unknown, interventions to improve CCA blood flow is possible to use the above mentioned neurochemicals to activate the DFA. Accumulation of knowledge from these and future investigations may provide insight into understanding the DFA mechanisms and developing therapeutic strategy in management of diseases involving CCA blood flow, such as migraine, Alzheimer’s disease, hypertensive disease and cerebral ischemia.

## References

[1] Gonzalez G, Onofrio BM, Kerr FWL (1975). Vasodilator system for the face.. J. Neurosurg..

[2] Lambert GA, Bogduk N, Goadsby PJ, Duckworth JW, Lance JW (1984). Decreased carotid arterial resistance in cats in response to trigeminal stimulation.. J. Neurosurg..

[3] Goadsby PJ, Lambert GA, Lance JW (1983). Effects of locus coeruleus stimulation on carotid vascular resistance in the cat.. Brain Res..

[4] Goadsby PJ, Piper RD, Lambert GA, Lance JW (1985). Effect of stimulation of nucleus raphe dorsalis on carotid blood flow.. II The cat. Am. J. Physiol..

[5] Wang MR, Kuo JS, Chai CY (2001). Nitric oxide produces different actions in different areas of the periaqueductal grey in cats.. Neurosci. Lett..

[6] Wang MR, Kuo JS, Chai CY (2002). Cardiovascular and vocalization reactions elicited by N-methyl-D-aspartate in the pretentorial periaqueductal grey of cats.. Clin. Exp. Pharmacol. Physiol..

[7] Kuo JS, Wang MR, Liu RH, Yu CY, Chiang BN, Chai CY (1987). Reduction of common carotid resistance upon stimulation of an area dorsal to the facial nucleus of cats.. Brain Res..

[8] Nakai M, Tamaki K, Ogata J, Matsui Y, Maeda M (1993). Parasympathetic cerebrovasodilator center of the facial nerve.. Circ. Res..

[9] Kuo JS, Chyi T, Yang MCM, Chai CY (1995). Changes in intra- and extra cranial tissue blood flow upon stimulation of a reticular area dorsal to the facial nucleus in cats.. Clin. Exp. Pharmacol. Physiol..

[10] Chyi T, Wang SD, Gong CL, Lin SZ, Cheng V, Kuo JS (2005). Preganglionic neurons of the sphenopalatine ganglia reside in the dorsal facial area of the medulla in cats.. Chin. J. Physiol..

[11] Lee TJF (2002). Sympathetic modulation of nitrergic neurogenic vasodilation in cerebral arteries.. Jp. J. Pharmacol..

[12] Lee TJF, Chang HH, Lee HC, Chen PY, Lee YC, Kuo JS, Chen MF (2011). Axo-axonal interaction in autonomic regulation of the cerebral circulation.. Acta Physiol..

[13] Gong CL, Lin NN, Kuo JS (2002). Glutamatergic and serotonergic mechanisms in the dorsal facial area for common carotid artery blood flow control in the cat.. Auton. Neurosci..

[14] Cheng FC, Kuo JS (1995). High-performance liquid chromatographic analysis with electrochemical detection of biogenic amines using microbore columns.. Chromatogr. B..

[15] Li HT, Chen WY, Liu L, Yang CS, Cheng FC, Chai CY, Kuo JS (1996). The dorsal facial area of the medulla in cats inhibitory action of serotonin on glutamate release in regulating common carotid blood flow.. Neurosci. Lett..

[16] Kuo JS, Li HT, Lin NN, Yang CS, Cheng FC (1999). Dorsal facial area of cat medulla 5-HT2 action on glutamate release in regulating common carotid blood flow.. Neurosci. Lett..

[17] Gong CL, Chiu YT, Lin NN, Lin SZ, Cheng FC, Kuo JS (2004). Inhibitory actions of serotonin on glutamate release in dorsal medulla suppressing systemic arterial pressure of cats.. Neurosci. Lett..

[18] Kuo JS, Lee TJF, Chiu YT, Li HT, Lin NN, Tsai TT, Gong CL (2008). Nitric oxide and glutamate in the dorsal facial area regulate common carotid blood flow in the cat.. Eur. J. Pharmacol..

[19] Kuo JS, Huang YP, Chiu YT, Lin NN, Cheng CC, Hung YW, Lee TJF, Gong CL (2009). Glutamate release upon purinergic action in the dorsal facial area of the medulla increases blood flow in the common carotid artery in cats.. Neuroscience.

[20] Dergacheva O, Griffioen K, Wang X, Kamendi H, Gorini C, Mendelowitz D (2007). 5-HT2 Receptor Subtypes Mediate Different Long-Term Changes in GABAergic Activity to Parasympathetic Cardiac Vagal Neurons in the Nucleus Ambiguus.. Neuroscience.

[21] Comet MA, Bernard JF, Hamon M, Laguzzi R, Sevoz-Couche C (2007). Activation of nucleus tractus solitarius 5-HT2A but not other 5-HT2 receptor subtypes inhibits the sympathetic activity in rats.. Eur. J. Neurosci..

[22] Jeggo RD, Kellett DO, Wang Y, Ramage AG, Jordan D (2005). The role of central 5-HT3 receptors in vagal reflex inputs to neurones in the nucleus tractus solitarius of anaesthetized rats.. J. Physiol..

[23] Langman NJ, Smith CG, Whitehead KJ (2006). Selective serotonin re-uptake inhibition attenuates evoked glutamate release in the dorsal horn of the anaesthetised rat in vivo.. Pharmacol. Res..

[24] Best AR, Regehr WG (2008). Serotonin evokes endocannabinoid release and retrogradely suppresses excitatory synapses.. J. Neurosci..

[25] Wang SJ, Wang KY, Wang WC, Sihra TS (2006). Unexpected inhibitory regulation of glutamate release from rat cerebrocortical nerve terminals by presynaptic 5-hydroxytryptamine-2A receptors.. J. Neurosci. Res..

[26] Kuo JS, Chyi T, Cheng V, Wang JY (1992). Immunocytochemical characteristics of dorsal facial area of the medulla in cats.. Soc. Neurosci. Abstr..

[27] Kubo T, Hagiwara Y, Endo S, Fukumori R (2002). Activation of hypothalamic angiotensin receptors produces pressor responses via cholinergic inputs to the rostral ventrolateral medulla in normotensive and hypertensive rats.. Brain Res..

[28] Reynolds DJ, Lowenstein PR, Moorman JM, Grahame-Smith DG, Leslie RA (1994). Evidence for cholinergic vagal afferents and vagal presynaptic M1 receptors in the ferret.. Neurochem. Int..

[29] Kaiser S, Wonnacott S (2000). Alpha-bungarotoxin-sensitive nicotinic receptors indirectly modulate [(3)H]dopamine release in rat striatal slices via glutamate release.. Mol. Pharmacol..

[30] Gong CL, Chiu YT, Lin NN, Cheng CC, Lin SZ, Lee TJF, Kuo JS (2006). Regulation of the common carotid arterial blood flow by nicotinic receptors in the medulla of cats.. Br. J. Pharmacol..

[31] Kuo JS, Leung YM, Lin NN, Lee TJF, Gong CL (2010). Nicotine stimulation of the medulla increases blood flow of the common carotid artery in cats.. Auton. Neurosci..

[32] Wall MJ, Dale N (2007). Auto-inhibition of rat parallel fibre-Purkinje cell synapses by activity-dependent adenosine release.. J. Physiol..

[33] Wall MJ, Dale N (2008). Activity-dependent release of adenosine.A critical re-evaluation of mechanism.. Curr. Neuropharmacol..

[34] Thomas T, Spyer KM (1999). A novel influence of adenosine on ongoing activity in rat rostral ventrolateral medulla.. Neuroscience.

[35] Barraco RA, O'Leary DS, Ergene E, Scislo TJ (1996). Activation of purinergic receptor subtypes in the nucleus tractus solitarius elicits specific regional vascular response patterns.. J. Auton. Nerv. Syst..

[36] Mosqueda-Garcia R, Tseng CJ, Appalsamy M, Beck C, Robertson D (1991). Cardiovascular excitatory effects of adenosine in the nucleus of the solitary tract.. Hypertension.

[37] Burnstock G  (1996). P2 purinoceptors historical perspective and classification.. Ciba. Found. Symp..

[38] Koles L, Furst S, Illes P (2007). Purine ionotropic (P2X) receptors.. Curr. Pharm. Des..

[39] Ralevic V, Thomas T, Burnstock G, Spyer KM (1999). Characterization of P2 receptors modulating neural activity in rat rostral ventrolateral medulla.. Neuroscience.

[40] Ralevic V (2000). P2 receptors in the central and peripheral nervous systems modulating sympathetic vasomotor tone.. J. Auton. Nerv. Syst..

[41] Shigetomi E, Kato F (2004). Action potential-independent release of glutamate by Ca2+ entry through presynaptic P2X receptors elicits postsynaptic firing in the brainstem autonomic network.. J. Neurosci..

[42] Guo ZL, Longhurst JC (2003). Activation of nitric oxide-producing neurons in the brain stem during cardiac sympathoexcitatory reflexes in the cat.. Neuroscience.

[43] Maisky VA, Datsenko VV, Moibenko AA, Bugaychenko LA, Pilyavskii AI, Kostyukov AI, Kalezic I, Johansson H (2003). NO-generating neurons in the medullary cardiovascular centers of rodents and carnivores.. Comp. Biochem. Physiol..

[44] Tseng CJ, Liu HY, Lin HC, Ger LP, Tung CS, Yen MH (1996). Cardiovascular effects of nitric oxide in the brain stem nuclei of rats.. Hypertension.

[45] Wu WC , Wang Y , Kao LS , Tang FI , Chai CY  (2002). Nitric oxide reduces blood pressure in the nucleus tractus solitarius a real time electrochemical study.. Brain Res. Bull..

[46] Horn T, Smith BE, McLaughlin BE (1994). Nitric oxide actions in paraventricular nucleus: Cardiovascular and neurochemical implications.. Am. J. Physiol..

[47] Hirooka Y , Kishi T , Sakai K , Shimokawa H , Takeshita A  (2003). Effect of overproduction of nitric oxide in the brain stem on the cardiovascular response in conscious rats.. J. Cardiovasc. Pharmacol. Ther..

[48] Martins-Pinge MC , Baraldi-Passy I , Lopes OU  (1997). Excitatory effects of nitric oxide within the rostral ventrolateral medulla of freely moving rats.. Hypertension.

[49] Patel KP, Li YF, Hirooka YA (2001). Role of nitric oxide in central sympathetic outflow.. Exp. Biol. Med..

[50] Gong CL, Chiu YT, Lin NN, Cheng CC, Kuo JS (2007). Regulation of common carotid arterial blood flow by nitrergic neurons in the medulla of cats.. Eur. J. Pharmacol..

[51] Gong CL, Leung YM, Huang YP, Lin NN, Hung YW, Lee TJF, Kuo JS (2010). Nicotine activation of neuronal nitric oxide synthase and guanylyl cyclase in the medulla increases blood flow of the common carotid artery in cats.. Neurosci. Lett..

